# Use of polyethyleneimine polymer in cell culture as attachment factor and lipofection enhancer

**DOI:** 10.1186/1472-6750-4-23

**Published:** 2004-10-15

**Authors:** Ajith R Vancha, Suman Govindaraju, Kishore VL Parsa, Madhuri Jasti, Maribel González-García, Rafael P Ballestero

**Affiliations:** 1 Departments of Chemistry and Biology, Texas A&M University-Kingsville, Kingsville, TX 78363, USA

## Abstract

**Background:**

Several cell lines and primary cultures benefit from the use of positively charged extracellular matrix proteins or polymers that enhance their ability to attach to culture plates. Polyethyleneimine is a positively charged polymer that has gained recent attention as a transfection reagent. A less known use of this cationic polymer as an attachment factor was explored with several cell lines.

**Results:**

Polyethyleneimine compared favorably to traditional attachment factors such as collagen and polylysine. PC-12 and HEK-293 cells plated on dishes coated with polyethyleneimine showed a homogeneous distribution of cells in the plate, demonstrating strong cell adhesion that survived washing procedures. The polymer could also be used to enhance the adherence and allow axonal outgrowth from zebrafish retinal explants. The effects of this coating agent on the transfection of loosely attaching cell lines were studied. Pre-coating with polyethyleneimine had the effect of enhancing the transfection yield in procedures using lipofection reagents.

**Conclusion:**

Polyethyleneimine is an effective attachment factor for weakly anchoring cell lines and primary cells. Its use in lipofection protocols makes the procedures more reliable and increases the yield of expressed products with commonly used cell lines such as PC-12 and HEK-293 cells.

## Background

Molecular cell biology experimentation often requires the culture of primary cells or immortalized cell lines. The most common substratum used in cell culture consists of a plastic dish that offers a negatively charged surface. A drawback of this technology is that some anchorage-dependent cell types do not produce sufficient amounts of positively charged extracellular matrix proteins, adhering only weakly to the plastic substratum. Pre-coating of the plastic surface with extracellular matrix proteins such as collagen, fibronectin, laminin, etc., usually enhances the attachment of these cell types [1,2]. Synthetic polymeric cations such as polylysine or polyornithine have also been used as attachment promoting factors in numerous studies [3,4].

Polyethyleneimine (PEI) is an organic polymer that has a high density of amino groups that can be protonated. At physiological pH, the polycation is very effective in binding to DNA and can mediate the transfection of eukaryotic cells [5]. The original PEI-based protocol has been used in our laboratory successfully for transfections with HEK-293 (human embryonic kidney) and PC-12 (rat pheochromocytoma) cells. However, lipofection using some of the last generation cationinc lipids, LipofectAMINE-2000 or LipofectAMINE-Plus (Invitrogen), yielded higher efficiencies of gene delivery, particularly with PC-12 cells (M. González-García and R.P. Ballestero, unpublished observations). Several modifications of the original PEI-based protocol have been described that improve the efficiency of transfection significantly, rivaling the lipofection protocols [6-10], but they have not been attempted yet in our laboratory. A notable difference, related to the attachment of the cells to the substratum, was observed during the transfection experiments. Whenever the cells were transfected by the original PEI-based protocol, the cells remained firmly anchored to the plates throughout histochemical procedures. HEK-293 and PC-12 cells are considered weakly anchoring cells [11-14], and many were detached from the plates during histochemical stainings of cells transfected by lipofection. This initial observation prompted us to study the attachment-enhancing properties of PEI and its effects on lipofection of eukaryotic cells. PEI has been used previously to coat surfaces to promote the attachment of neurons in primary cell cultures [15,16]. A modified version of PEI that incorporates hydrophobic groups was demonstrated to be highly effective in the attachment of several cell lines, allowing differentiation of neurons and preventing cell loses during multiple washing steps [17].

This report presents a comparison of PEI with other traditional coating agents as attachment factors for several cell lines and primary cultures of retina tissue. Additionally, an enhancement of the transfection yield of weakly anchoring cell lines is shown by the combination of PEI coating with lipofection.

## Results

### PEI promotes the attachment of weakly anchoring cells and primary tissues

**Figure 1:**
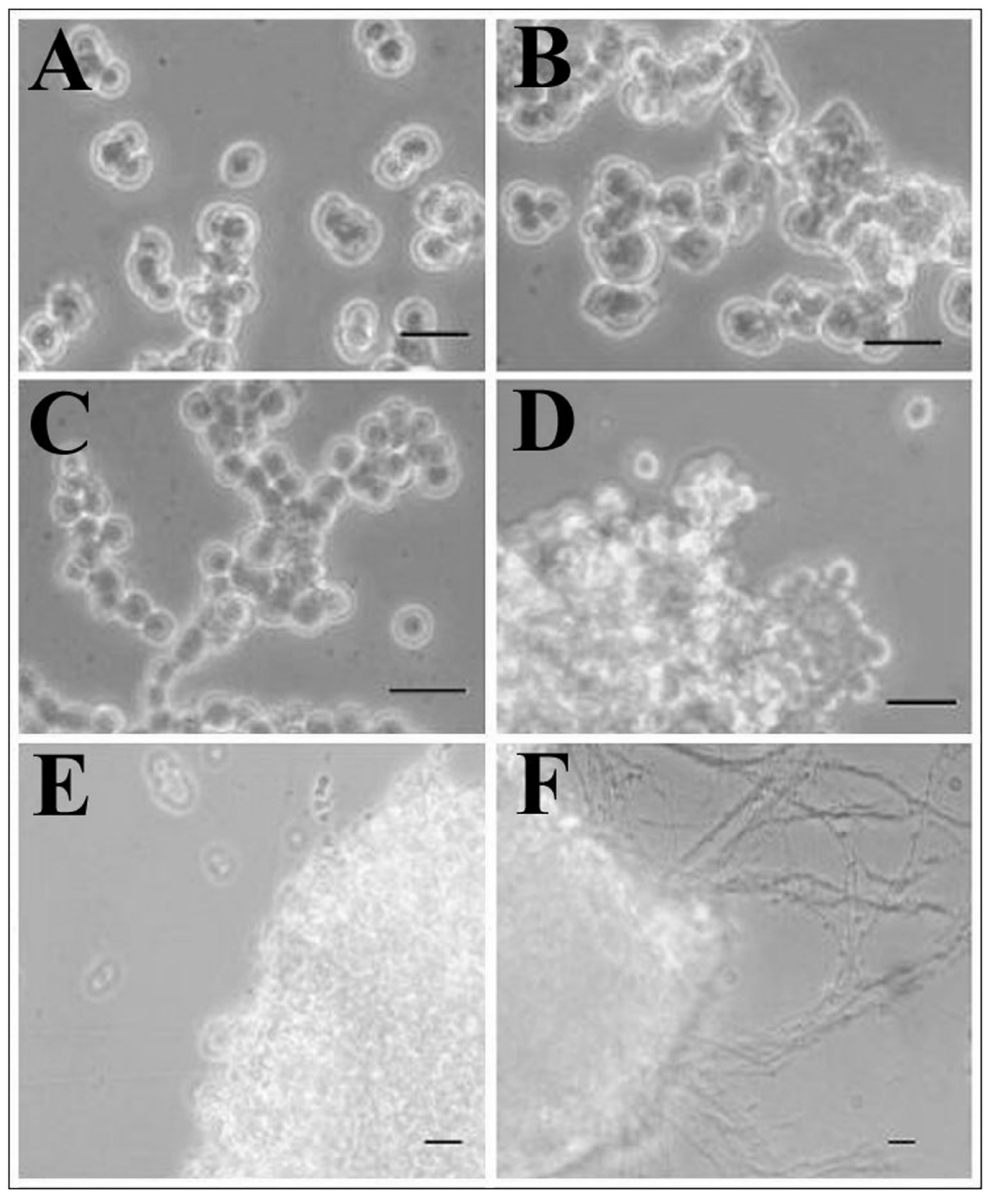
**Attachment of PC-12 cells and neuronal explants to culture dishes. **PC-12 cells attached better to plastic surfaces that were pretreated with anchoring-promoting factors, such as PEI (panel A), collagen (panel B), or PDL (panel C), as compared to the untreated plastic surface control (panel D), which contained cells in clusters and very loosely attached to the substratum (pictures were taken midway between center and edge of the plate). PEI coating resulted in a homogeneous distribution of cells firmly attached to the dish substratum (panel A). PEI promotes attachment to culture dishes of retinal explants from zebrafish (panel E). This attachment factor was supportive of neurite extension in retinal explants primed for axonal growth by a pre-conditioning lesion to the optic nerve (panel F). The bar in the photomicrographs corresponds to 25 μm in panels A-D, 50 μm in panel E, and 100 μm in panel F.

PC-12 cells are used as a model of neuronal differentiation in the laboratory, as treatment of these cells with nerve growth factor (NGF) induces neurite extension and the expression of biochemical markers of the sympathetic neuronal phenotype [18]. They grow as weakly anchoring cells and collagen or polylysine polymers are frequently used for pre-coating plates for PC-12 cell culture [13,19,20]. PC-12 cells were cultured in the laboratory in naive wells or in wells pre-coated with diverse attachment factors (multiwell-12 tissue culture dishes), to observe the effect on the anchorage of the cells to the substratum. In the absence of any coating agent, the cells showed a characteristic tendency to form clusters of cells that accumulate towards the center of the well; these cells are firmly attached among themselves, but very weakly attached to the tissue culture dish (Figure 1D). This results in a very heterogeneous distribution of cells (very few cells remain towards the edge of the wells) and significant loss of cells during washing procedures or changes of medium. Pretreatment of the plates with PEI resulted in a significantly more homogeneous distribution of cells in the well, with cells attaching to the plate firmly, showing a much lower tendency to clustering (Figure 1A). For comparison, plates were also pretreated with other commonly used coating agents, collagen (Figure 1B) and poly-D-Lysine (PDL; Figure 1C), resulting also in firmer attachment of the cells to the plates and a more homogeneous distribution of cells.

To test further the anchoring enhancement property observed with the PEI pretreatment of the culture dishes, a second system was utilized. Retinal explants from teleost fish have been used in the study of nerve regeneration [21,22]. When a lesion is applied to the optic nerve of the fish, a regeneration response is initiated and the retinal ganglion cells (RGCs) of the retina re-extend their axon towards the tectal target tissue. If the retina from such a "primed" fish is explanted and cultured, the regeneration response is observed *in vitro *by the accelerated extension of long neurites. However, this phenomenon requires the use of extracellular matrix or attachment factors (for example collagen or PDL coating), as the explants show very low affinity for the uncoated plastic surface. Experiments were conducted to observe if PEI could act as an attachment factor conducive to axonal outgrowth from zebrafish retinal explants. Retinal explants from control zebrafish eyes were able to attach to PEI-pretreated culture dishes (Figure 1E). Furthermore, retinal explants from fish that received a conditioning lesion were able to extend axons vigorously when cultured using PEI as attachment factor (Figure 1F).

**Figure 2:**
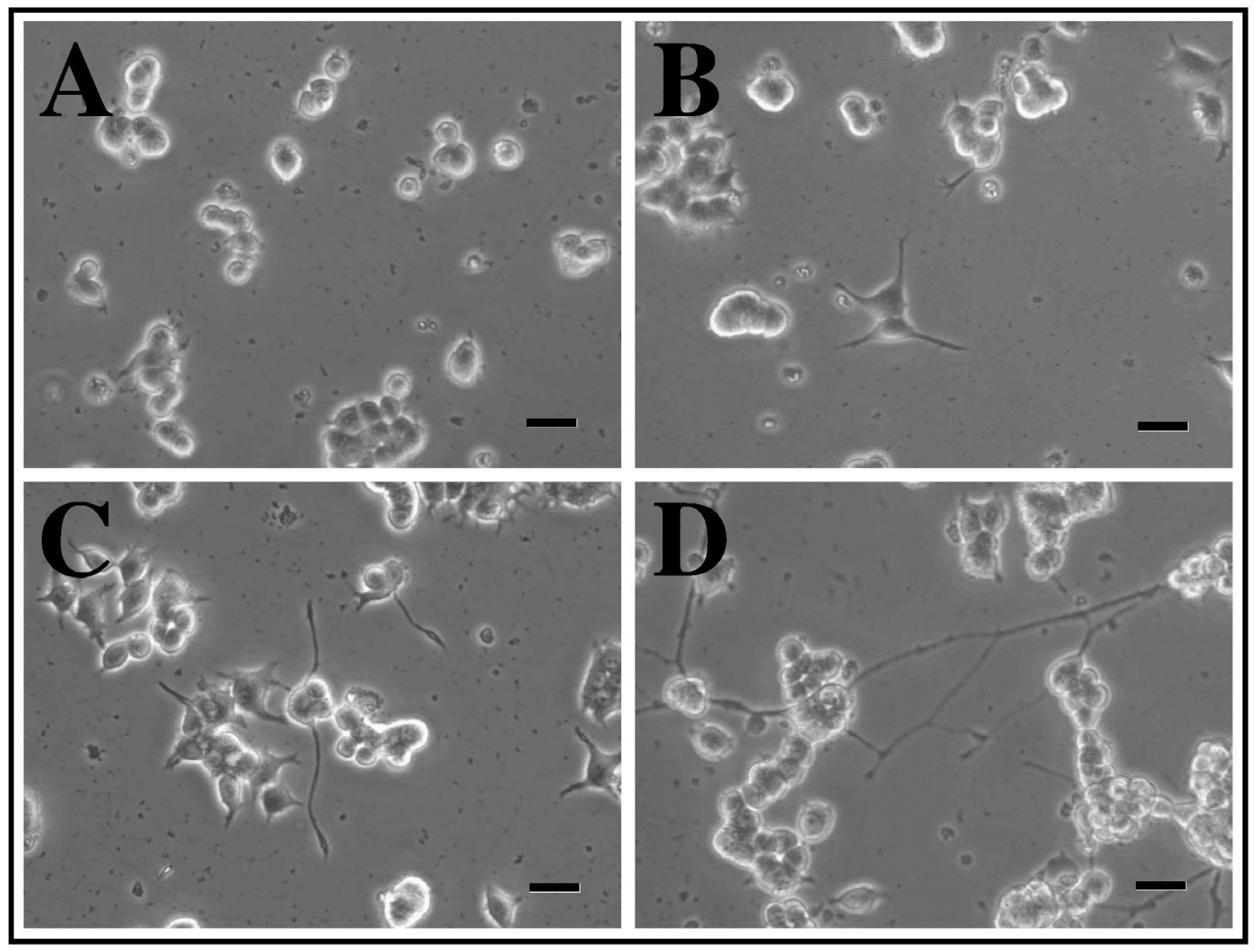
**Differentiation of PC-12 cells attached to culture dishes coated with PEI. **PC-12 cells attached to PEI coated dishes remained firmly anchored to the plate and extended neurites upon treatment with NGF. The pictures show the progress of the differentiation of the treated cells over time: 0 h (panel A), 24 h (panel B), 48 h (panel C) and 96 h (panel D) after the initiation of treatment with 100 ng/ml of NGF. The bar in the photomicrographs corresponds to 25 μm.

The results with the retina explants suggested that neuronal cells attached to PEI-coated dishes can differentiate, generating neurites that attach well to the substrate. To test this hypothesis with the pro-neuronal PC-12 cells, differentiation experiments by NGF treatment were performed with cells attached to dishes coated with PEI. The PC-12 cells treated with NGF remained firmly attached to the plate over several days, generating networks of neurites (Figure 2). The results indicate that PEI is permissive for the differentiation process and for the attachment of the neurites to the dish. The differentiated cells remained firmly anchored throughout immunocytochemistry experiments (M. Challa, G. R. Chapa, M. González-García and R. P. Ballestero, unpublished results).

### Strength of anchoring of eukaryotic cells to culture dishes pretreated with PEI and other attachment factors

**Figure 3:**
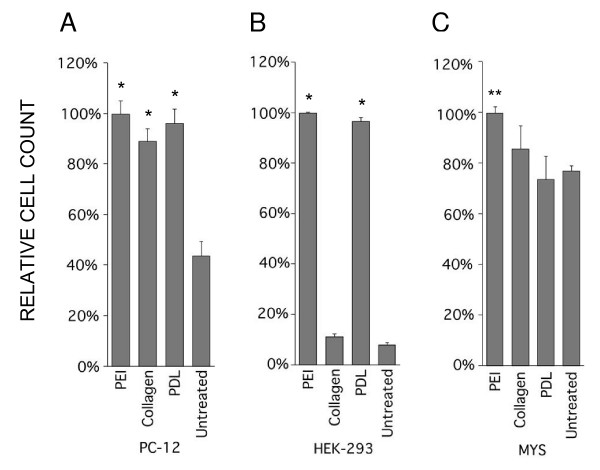
**PEI coating promotes firm attachment of weakly anchoring cells to the substratum. **The loss of cells induced by a protocol that involved multiple washings was measured to assess the strength in the attachment of cells to culture dishes coated with factors that promote cell anchoring. PEI was compared to other attachment factors (collagen, PDL) and to untreated control dishes. The relative cell counts were normalized by assigning an arbitrary value of 100.0% to the PEI-pretreated dishes. PEI coating resulted in significant enhancement of anchoring strength in PC-12 cells (graph A) and in HEK-293 cells (graph B), comparing favorably to other commonly used attachment factors. No benefits were observed in the attachment of a strongly anchoring cell line (MYS cells, graph C). The bars show the average ± standard deviation of triplicate assays, which are representative of at least three different experiments performed (* significantly higher than untreated control, p < 0.01; ** significantly higher than untreated control in the experiment presented, p < 0.01, but significance not maintained in independent experiments).

Three different cell lines were selected to test the ability of PEI to promote strong anchoring to plastic culture dishes. PC-12 and HEK-293 cells have been described above as weakly anchoring. On the other hand, MYS cells are primary fibroblasts that adhere strongly to plastic culture dishes, growing to form a monolayer of cells on the surface. To test the strength of anchoring of the diverse cells to plates, a protocol was performed in which 4 consecutive washes with isotonic buffer were performed, followed by the use of a colorimetric protocol for counting the cells that remained in the plate (based on the vital dye neutral red). Plates pretreated with the diverse attachment factors were compared with plates that received no pretreatment (untreated). Experiments were performed in triplicate, and the averages of dye retained in the plates that received each treatment were calculated. Those averages were normalized to the average obtained with the PEI pretreatment, which was assigned the arbitrary value of 100.0% in all the experiments. The results from representative experiments with the 3 cell lines are shown in Figure 3 (at least three independent experiments were conducted with each cell line). PC-12 cells attached almost equally well to plates coated with PEI, collagen or PDL (relative cell counts of 100.0% ± 5.3%, 89.3% ± 5.0% and 96.3% ± 5.8%, for PEI, collagen and PDL respectively). However, a significant loss of cells was observed when comparing untreated plates with PEI-pretreated plates, with a relative cell count of 43.9% ± 5.8% (Figure 3A). In the case of HEK-293, the cells attached strongly to both PEI- and PDL-pretreated wells. In the representative experiment shown in Figure 3B, the relative counts with those two treatments were 100.0% ± 0.4% and 96.8% ± 1.7% respectively. However, a large number of cells were lost when plated in the untreated wells (relative count of 8.3% ± 0.6%), or in the wells that were pretreated with collagen (11.5% ± 1.2%), suggesting that these cells attach fairly loosely to plastic or to collagen-coated wells. Finally, when the fibroblast cells (MYS cells) were utilized, the cells seemed to attach rather well to all four surfaces, including to the untreated wells (Figure 3C). Figure 3C shows a representative plot, displaying relative MYS cell counts of 100.0% ± 2.2%, 85.9% ± 7.8%, 73.7% ± 6.6%, and 77.1% ± 1.4%, with wells pretreated with PEI, collagen, or PDL, or untreated wells respectively. This cell line therefore attaches fairly well to the untreated plastic surface comparatively to the PC-12 and HEK-293 cell lines.

To provide an indication of the variation among experiments, the averages ± standard deviations of the relative cell counts obtained in the independent experiments were calculated for each cell line and treatment (note that since in all the experiments the count for PEI-pretreated wells was set at 100.0%, the value for the global average with this coating agent is exactly 100.0% for all the cell lines). The comparative results for the other treatments are indicated below. For the PC-12 cells, the relative counts were 81.5% ± 8.8% for collagen-pretreated wells (n = 4), 93.9% ± 21.2% for PDL-pretreated wells (n = 4), and 52.1% ± 13.7% for untreated wells (n = 4). For the HEK-293 cells, the relative counts were 16.3% ± 12.7% for collagen-pretreated wells (n = 3), 99.6% ± 2.5% for PDL-pretreated wells (n = 3), and 9.0% ± 4.1% for untreated wells (n = 3). In the experiments with MYS cells, the average of the relative counts with collagen-pretreated wells was 92.7% ± 16.2% (n = 3), 71.1% ± 6.7% for PDL-pretreated wells (n = 3), and 78.4% ± 11.5% for untreated wells (n = 3). Statistical analysis indicates that the enhancement of adhesion of both PC-12 and HEK-293 cells to PEI-pretreated dishes versus untreated plastic is significant (p < 0.05 and p < 0.01 respectively, t-test analysis), while it is not statistically significant for MYS cells (p > 0.05).

**Figure 4:**
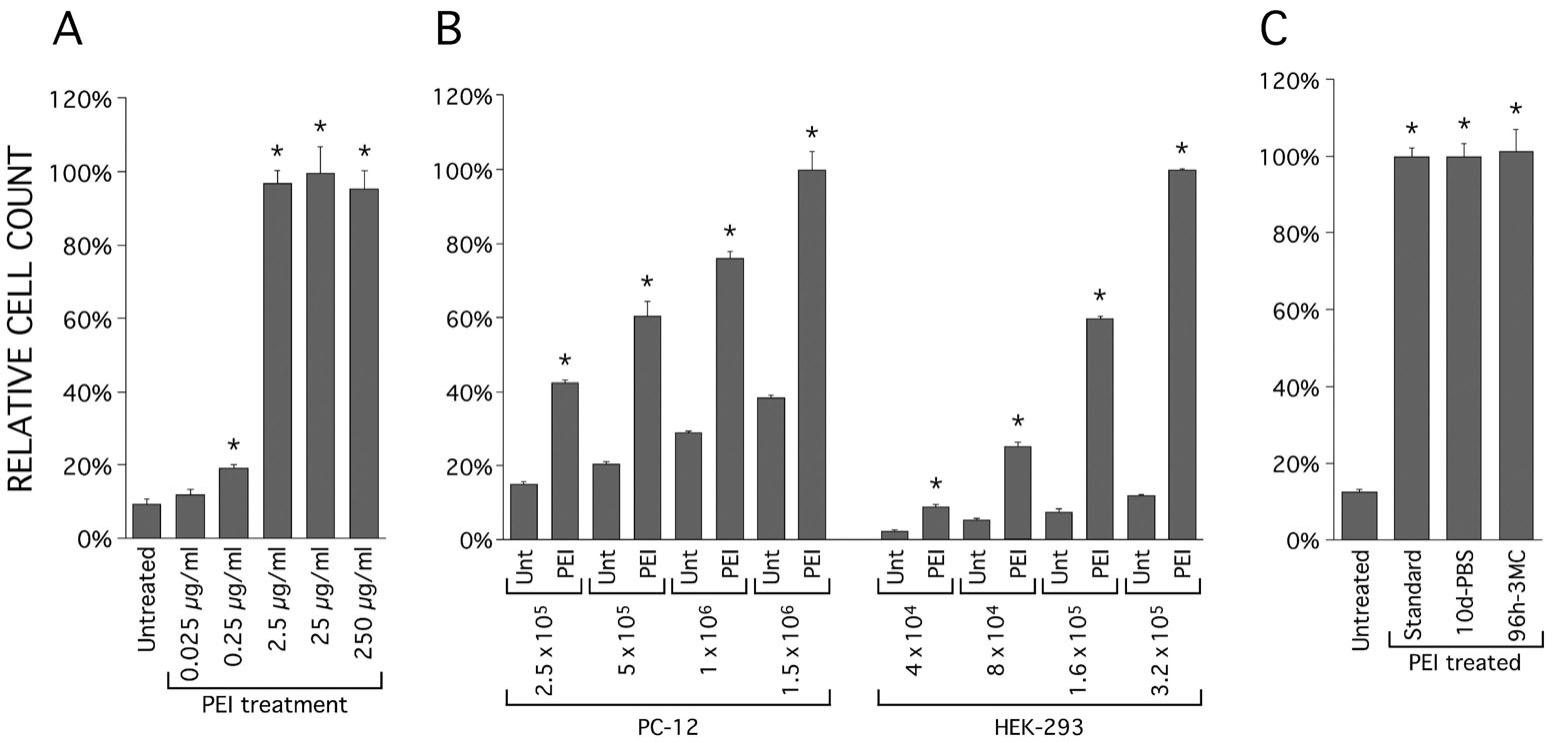
**Characterization of the properties of PEI as an attachment factor. **Graph A: Increasing doses of PEI were tested to determine the concentration range for optimal coating. PEI showed full attachment enhancement at concentrations of 2.5 μg/ml and higher. Graph B: PEI promoted attachment of both PC-12 and HEK-293 cells over a wide range of cell numbers (numbers tested are indicated in X axis). Graph C: PEI coating remained stable onto the culture dish surface over long incubation periods and medium changes (10d-PBS: PEI-treated dish incubated for 10 days in PBS at 4°C; 96 h-3MC: PEI-treated dish incubated for 96 h with 3 medium changes at 37°C). For all the graphs, the bars show the average ± standard deviation of triplicate assays, which are representative of at least 3 independent experiments (* significantly higher than untreated control, p < 0.01).

To further characterize the properties of PEI as an attachment factor for weakly anchoring cells, experiments were carried out to analyze the dosage of PEI that can provide optimal cell anchoring, the range of cell numbers that can benefit from the presence of PEI, and the stability of PEI as attachment factor. Figure 4A shows the results from a representative experiment testing the strength of attachment of HEK-293 cells to dishes coated with various doses of PEI. The cell counts were normalized to the value obtained for the treatment with 25 μg/ml of PEI, which was set at 100.0%. The results indicate that concentrations of PEI of 2.5 μg/ml or higher resulted in maximal attachment enhancement, suggesting that the surface of the plastic dish is fully coated with the polymer at these concentrations. The higher concentrations of the polymer did not seem to have toxic effects on the cells if the excess of PEI remaining in solution was removed thoroughly (slight toxic effects were observed at the 250 μg/ml concentration if the solution was not fully removed after the treatment). The experiment shown is representative of 4 independent experiments. Figure 4B represents the results obtained with various numbers of PC-12 and HEK-293 cells. The figure shows relative cell counts, where an arbitrary value of 100.0% was assigned to the average count obtained from the PEI-treated wells with the highest number of each cell line (3.2 × 10^5 ^HEK-293 cells and 1.5 × 10^6 ^PC-12 cells). The results indicate that PEI worked well as an attachment factor over a wide range of cell numbers for both PC-12 and HEK-293 cells. Lower cell numbers could not be tested reliably because they were close to the limit of sensitivity of the neutral red assay. The results shown are representative of at least 4 independent experiments performed with each cell line. Figure 4C shows the results of an experiment conducted to test the stability of PEI as an attachment factor. In this experiment, a set of plates were treated with PEI and then kept in PBS at 4°C for 10 days. In a second test, plates were treated with PEI and kept (with medium) in the 37°C CO_2 _incubator for 3 days, with a medium change performed every 24 h. The performance of PEI in these plates was then compared with plates treated with PEI using the standard protocol described for previous experiments. The results show relative cell counts normalized to the average of the absorbances obtained with the standard PEI-treated plates, which was given the arbitrary value of 100.0%. The results indicate that the PEI coating remains stable on the surface of the plastic dish for at least 10 days of refrigeration, and that it is not removed by incubation at 37°C in medium or even by repeated medium changes. Results are representative of 4 independent experiments performed in triplicate.

### PEI pretreatment enhances lipofection of weakly anchoring cells

**Figure 5:**
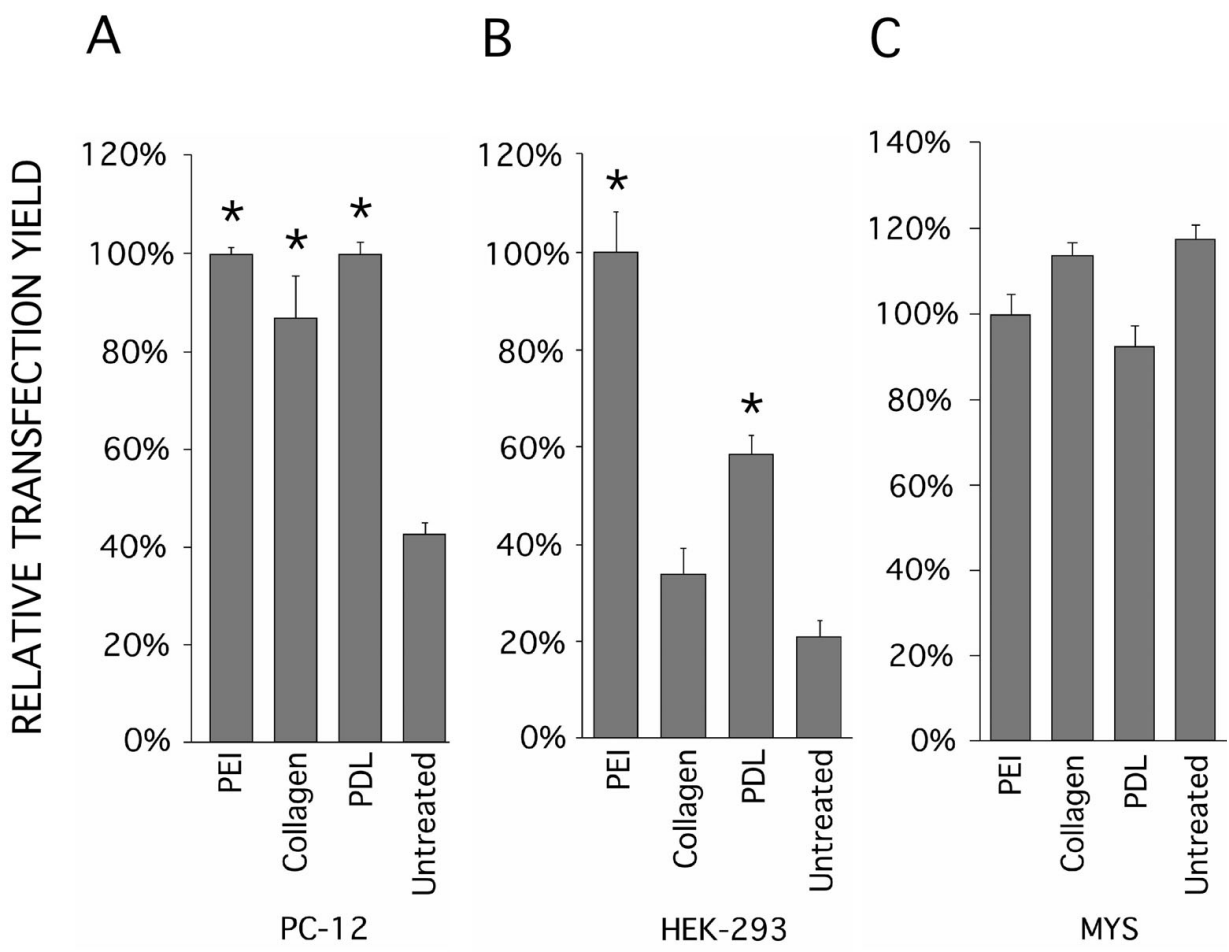
**PEI enhances transfection of weakly anchoring cells. **Transfection yields were determined by β-galactosidase assays and normalized to the yields obtained with PEI coating of the culture dish (to which it was assigned an arbitrary value of 100.0%). PEI and other attachment factors enhanced the transfection of loosely attaching cell lines such as PC-12 cells (graph A) and HEK-293 cells (graph B). No significant effect was observed with tightly attaching cells such as MYS fibroblasts (graph C). The bars show the average ± standard deviation of triplicate assays, which are representative of at least two independent experiments (* significantly higher than untreated control, p < 0.01).

Transfection of eukaryotic cells by lipofection involves several steps, media additions and replacements, which can take a toll in weakly anchoring cells. Even if care is practiced to avoid cell loses, the weakly anchored cells may be less efficient in the uptake of the transfection complexes. Since PEI pretreatment of cell culture dishes increased the strength of attachment of cells to such surfaces, it was hypothesized that the attachment factor may have a positive effect in transfection yields obtained by lipofection. The three cell lines described above were used to test this hypothesis utilizing the coating agents previously examined. Transfections were performed with a plasmid encoding the reporter enzyme β-galactosidase. The transfection yield was monitored by determination of reporter enzyme activity in lysates from the transfected cells. At least two independent assays in triplicate were performed with each cell line. Representative plots are shown in Figure 5. The yields (β-galactosidase activities) were normalized to the activity obtained with PEI pre-coating, which was set as a reference at 100.0%. Generally, it was observed that the transfection yields were improved in the weakly anchoring cells by the pre-coating with agents that promote cell attachment to the plate. In the case of PC-12 cells, pre-coating of wells with PEI, collagen or PDL resulted in a 2- to 3-fold enhancement of transfection yield related to the untreated wells: relative yields of 100.0% ± 1.4% for PEI, 87.0% ± 8.7% for collagen, and 100.1% ± 2.4% for PDL, versus 42.9% ± 2.2% for the untreated wells (Figure 5A). The improvement of transfection yield by PEI pretreatment was more pronounced for HEK-293 cells. The experiment in Figure 5B shows a relative activity of 21.1% ± 3.4% for the cells in the untreated wells, when compared with 100.0% ± 8.4% for the PEI-pretreated wells (approximately 5-fold increase). Pretreatment with collagen or PDL resulted in more modest inductions (approximately 2- to 3-fold in Figure 5B). The lowest improvement was observed with collagen, similarly to the lower enhancement of attachment of HEK-293 cells that was previously observed in Figure 3B. Finally, for the strongly attaching MYS fibroblasts, there was not a significant positive effect observed by using attachment factors in the transfection procedure. Variations were usually less than 20% for all the experimental conditions. The representative experiment shown in Figure 5C in fact shows a slightly higher transfection yield for the untreated wells than for the PEI-pretreated plates (relative activity of 117.7% ± 3.2% for the untreated wells versus 100.0% ± 4.8% for the PEI-pretreated wells, or approximately 1.2-fold higher transfection yield in the control).

Compiling the results from all the experiments performed, the average fold-induction (± standard deviation) observed in the transfection yield of PC-12 cells anchored to PEI as compared with cells on untreated wells was 2.4-fold (± 0.1-fold; n = 3), while the enhancement with HEK-293 cells was 6.3-fold (± 0.5 fold; n = 2). t-test statistical analysis indicates that both increases are significant (p < 0.05). No significant transfection enhancement was observed in the experiments with MYS cells, with an average fold-change of 1.0-fold (± 0.3-fold; n = 2) by the pretreatment with PEI (basically identical to the yield without treatment).

## Discussion

While some cell lines are able to produce extracellular matrix components in sufficient quantities to allow them to attach well to plastic culture dishes, some others have a more limited capacity, resulting in cells that are loosely attached to the dish. Natural extracellular matrix factors such as collagen, laminin, fibronectin, etc., can be utilized in the culture of these cells [2] and in the performance of multiple laboratory procedures that require the cells to remain attached to the culture vessel, for example immunocytochemical procedures. Other more economical alternatives have been developed, such as the use of polylysine or polyornithine [3,4]. A much less utilized polymer, PEI, offers a successful alternative. The most economical of all the mentioned factors, PEI seems to perform the task of attachment factor efficiently with primary neurons (see Figure 1 and references [15,16]), and with weakly anchoring cell lines (see Figures 1, 2 and 3, and reference [17]). The PEI solution is very easy to prepare, it can be frozen for several months without any loss of activity, and remains active on the plastic surface for numerous days after the treatment (Figure 4C). The anchoring effect of PEI seemed to work well for a wide range of cell numbers for both PC-12 and HEK-293 cells (see Figure 4B). PEI was efficient with high numbers of PC-12 cells probably by preventing cell-cell interactions (that result in the "clumping" of the cells), favoring instead the attachment to the dish surface. PEI worked well with HEK-293 cells in the range of 4 × 10^4 ^to 3.2 × 10^5 ^cells per well. When larger numbers of HEK-293 cells were utilized the results were more variable, suggesting that when these cells are near confluency they may be able to form monolayers that attach more firmly to the plate, however further experiments are necessary to study this hypothesis. The effects of PEI and the other cationic attachment factors on PC-12 and HEK-293 cells suggest that these cells have a limited capacity to produce an effective extracellular matrix to allow them to attach to the plastic surface, resulting in weak anchoring to the dishes on their own. Cationic polymers such as PEI and PDL seem to work very effectively to coat the surface of the dish and substitute for the absence of the extracellular matrix components. On the other hand, fibroblast cells (such as the MYS cells) are very efficient producing extracellular matrix proteins, which allows them to attach firmly to the culture dishes, therefore no benefit was observed by the use of attachment factors with these cells (see Figure 3).

Lipofection is an efficient transfection procedure that requires culture dish manipulations and media changes. This fact causes difficulties when using cells that are not well anchored to the culture dish. The experiments presented support the idea that attachment factors can enhance the yield of lipofection of weakly anchoring cells. PEI provided very good results with PC-12 and HEK-293 cells, while the other factors tested showed more specific and limited effects (see Figure 5). Based on the results regarding the anchoring promotion effect of PEI and the other factors, it is likely that the increases in the yields of β-galactosidase observed are due to a larger number of cells remaining in the dishes that were treated with attachment factors. However, the transfection procedures were performed trying to minimize the cell loses, therefore it is also possible that part of the effect observed could be derived from a higher transfection efficiency of the cells that are more firmly attached versus the cells that are loosely attached or forming clusters. Cells in suspension are often harder to transfect than cells anchored to the substratum [9,10]. Preliminary experiments in which the transfection yield was normalized to the amount of protein in the lysate suggested that the increased yield is due primarily to the larger number of cells rather than an increase in efficiency (S. Govindaraju, K.V.L. Parsa, M. González-García and R.P. Ballestero, unpublished results). Future experiments will analyze these possibilities in more detail. In any case, the PEI pretreatment offers additional advantages, for example the firmly attached cells can be used in immunocytochemical and immunofluorescence analysis with great ease and virtually no cell loss (data not shown). Most protocols of transfection of PC-12 cells suggest the use of an attachment factor, however this is usually not the case for protocols involving the widely used HEK-293 cells [19,20,23,24]. Our experiments suggest that pretreatment of culture surfaces with PEI is advantageous in lipofection protocols with both cell lines.

## Conclusions

PEI is used frequently as a transfection reagent. A second application of this reagent as an attachment factor is much less recognized. A comparison of PEI with two very commonly used attachment factors for cell and tissue culture showed that PEI is highly efficient and convenient. Two commonly used cell lines, PC-12 and HEK-293, attached firmly to plastic dishes coated with PEI. Although the anchoring properties of PEI had been previously recognized, its use with these two cell lines had not been characterized in detail in comparison with other frequently used coating agents. PEI will likely work with a variety of weakly anchoring cells, for example PEI was shown to be effective with primary retinal explants in this report. Additionally, the results presented indicate that the use of coating agents can enhance lipofection protocols. Cell culture and transfection protocols using PC-12 cells frequently involve the use of coating agents (commonly collagen or polylysine), but this is not so for protocols with HEK-293 cells. PEI was very effective in improving lipofection protocols with both cell lines. The firm attachment afforded by PEI allowed transfections with cationic lipids (lipofection) to provide higher yields and more consistent results.

## Methods

### Tissue culture and microscopy

PC-12 (rat pheochromocytoma, ATCC CRL-1721) cells were cultured with RPMI complete medium: RPMI medium (Invitrogen) supplemented with 10% horse serum and 5% fetal bovine serum (both from Hyclone), 100 u/ml of penicillin, 100 μg/ml of streptomycin, 10 mM HEPES, 2 mM glutamax and 1 mM sodium pyruvate (all from Invitrogen). HEK-293 (293, human embryonic kidney, ATCC CRL-1573) and MYS (MYS-Cl-2-BCF1, mouse yolk sac, ATCC CRL-9292) cells were cultured in DMEM complete medium: DMEM medium (Invitrogen) supplemented with 10% fetal bovine serum (Hyclone), 100 u/ml of penicillin, 100 μg/ml of streptomycin and 2 mM glutamax (all from Invitrogen). Cell lines were maintained at 37°C in a 5% CO_2 _atmosphere in a tissue culture incubator (NuAire). Zebrafish retinal explants were maintained in L-15 complete medium: L15 medium (Sigma) supplemented with 1% fetal bovine serum (Hyclone), 100 u/ml penicillin, 100 μg/ml of streptomycin and 2 mM glutamax (all from Invitrogen). The explants were incubated at 28°C at normal atmospheric CO_2 _concentrations.

For the culture and microscopic observation of PC-12 cells, the wells of a multiwell-12 tissue culture dish (MW12; Nunclon) were pretreated for 20 min with 100–200 μl of the different solutions of attachment factors: PEI-800 kDa, from Fluka, at 25 μg/ml in 150 mM NaCl; PDL, from ICN, at 100 μg/ml in deionized water; bovine dermal collagen, from ICN, 3 mg/ml aqueous solution. Usually, each column of wells in the MW12 was pretreated with one of the attachment factors, while the last column was left untreated. After removal of the different attachment factor solutions, 1 ml of a PC-12 cell suspension (approximately 0.5 × 10^6 ^cells) was added to each well of the MW12 and the plate was incubated at 37°C in a 5% CO_2 _atmosphere for 48 h. The cells were then examined under a phase contrast Olympus-CK40 microscope and photomicrographs were obtained with a Pixera Penguin 150 CL camera. The experiment was performed in triplicate and pictures were taken at the center, near the edge and midway between center and edge of the plate, to evaluate the distribution of cells on the wells.

For the microscopic observation of retinal explants, optic nerve crush (conditioning lesion) was performed retro-orbitally with anesthetized zebrafish 10 days prior to explantation, using protocols previously described [21,25]. Control explants were obtained from unoperated fish. The retinal pieces were laid on the wells of a MW12 that were pretreated with PEI as described above, and cultured in 200 μl of L15 complete medium at 28°C for 3 days. Photomicrographs were obtained as previously described.

### PC-12 differentiation with NGF

The wells of a MW12 were pretreated with PEI as described above. The wells were then seeded with 2.5 × 10^5 ^PC-12 cells and cultured with RPMI complete medium as previously described. After 24 h, the medium was replaced by RPMI medium with low serum (same as RPMI complete medium, except for lower concentrations of sera: 1% horse serum and 0.5% fetal bovine serum), which was supplemented with NGF (Sigma) at 100 ng/ml to induce differentiation. The culture medium was replaced by new RPMI low serum medium with 100 ng/ml of NGF every two days. At different times during the differentiation process (0, 24, 48, and 96 h after the initial NGF addition), photomicrographs were obtained as previously described.

### Cell number measurement with neutral red dye

Pretreatment of MW12 dishes with the attachment factors was performed as described above. PC-12, HEK-293 and MYS cells were counted using a Neubauer hemocytometer, and the MW12 plates were seeded with 1 × 10^6 ^cells per well for the experiments with PC-12 cells, 1.5 × 10^5 ^cells for the experiments with HEK-293 cells, or 3 × 10^4 ^cells for the experiments with MYS cells. The MW12 dishes were incubated at 37°C in a 5% CO_2 _atmosphere for 48 h in the case of PC-12 cells, or 24 h for HEK-293 and MYS cells. In all the experiments, the wells were washed 4 times with phosphate buffered saline (PBS) to test the strength of anchorage to the substratum. To estimate the number of cells remaining after the multiple washings, a procedure that utilizes the vital dye neutral red was employed [26]. Briefly, the cells were incubated for 90 min at 37°C with 750 μl of a solution of 0.01% neutral red in PBS, and then the excess dye was removed with two washes in PBS. The dye retained by the cells was extracted with 900 μl of ethanol-citrate solution (1:1 mixture of 0.1 M citrate pH 4.2 solution and ethanol) for 20 min with gentle agitation. Relative cell counts were determined by measuring the amount of dye spectrophotometrically at 540 nm (Beckman DU500 spectrophotometer). All experiments were performed in triplicate. To compare the experiments, absorbances were normalized to the values obtained from the PEI-pretreated wells.

### Statistical analyses

Two-sample (unpaired) t-test analyses were performed with a Microsoft Excel worksheet designed for this purpose. The worksheet calculates the t-factor by comparing 2 independent sets of data and estimates the probability (p) of obtaining that result assuming that the two samples came from the same population (null hypothesis: mean-1 = mean-2; alternate hypothesis: mean-1 < > mean-2) based on the Student's t-distribution (two-tailed). The averages of the two samples from the same experiment were considered statistically different whenever p was lower than 0.01. For comparisons of the independent experiments, one-sample t-test analyses were utilized to estimate the p values and assess the statistical significance of the difference observed between the PEI-treated wells and the untreated controls. The effect of PEI was considered significant whenever p was lower than 0.05.

### Determination of effective PEI dosages

For the determination of optimal concentrations of PEI for the pretreatment procedure, a series of solutions of PEI were prepared so that their final concentrations of PEI polymer (w/v) were 0.025, 0.25, 2.5, 25 and 250 μg/ml. They were used to coat MW12 tissue culture dishes as previously described for the 25 μg/ml solution. To test the efficacy of coating of the solutions, 2 × 10^5 ^HEK-293 cells were seeded onto the dishes and incubated at 37°C for 24 h. Untreated wells were seeded in the same manner as controls.

The dishes were then subjected to the multiple washing procedure described above and the number of cells remaining in the dishes was measured by the neutral red dye procedure as described previously. All experiments were performed in triplicate and the relative cell counts were calculated by normalization to the values obtained with 25 μg/ml of PEI, which was assigned a value of 100.0%.

### Determination of effective cell number ranges for PC-12 and HEK-293 cells

The wells of MW12 culture dishes were pretreated with 25 μg/ml of PEI as described above. The wells were then seeded with various numbers of either HEK-293 cells (4 × 10^4^, 8 × 10^4^, 1.6 × 10^5 ^and 3.2 × 10^5^) or PC-12 cells (2.5 × 10^5^, 5 × 10^5^, 1 × 10^6 ^and 1.5 × 10^6^). Control untreated MW12 culture dishes were seeded with the same numbers of cells in parallel with the treated plates. After 24 h for the HEK-293 cells or 48 h for the PC-12 cells, the strength of anchoring of the cells to the dishes was measured as described before and relative cell numbers were determined (normalized to the raw absorbance values obtained from the PEI-pretreated wells with the highest numbers of each cell line tested, 3.2 × 10^5 ^for HEK-293 cells and 1.5 × 10^6 ^for PC-12 cells, which were assigned arbitrarily a value of 100.0%). All experiments were performed in triplicate.

### Stability of PEI coating experiments

To analyze the stability of the PEI attachment factor onto the plastic surface of the tissue culture dish, two tests were performed. One MW12 dish was coated with 25 μg/ml of PEI as previously described, then the PEI solution was replaced by PBS and the dish was kept at 4°C for 10 days. A second MW12 dish was coated with 25 μg/ml of PEI, then the PEI solution was replaced by DMEM complete medium and the dish was kept at 37°C in the CO_2 _incubator for 24 h. Afterwards, the medium was replaced by fresh DMEM complete medium and incubated for a further 24 h. This process was repeated for a third time, so that the dish was incubated for a total of 96 h with 3 medium changes. These two dishes were then seeded with 2 × 10^5 ^HEK-293 cells, in parallel with a third MW12 in which a column of wells was pretreated with PEI using the standard protocol described earlier (an untreated column of wells in this dish was used for the controls). The strength of attachment of cells to these dishes was determined as mentioned before, and relative cell counts were calculated by normalization to the value obtained with the standard PEI-treated wells, which was assigned arbitrarily a value of 100.0%. All experiments were performed in triplicate.

### Transfection yield determinations

MW12 culture dishes were pretreated and seeded with cells as indicated above for the cell number measurements. The cells were subjected to lipofection with 1 μg per well of the plasmid pcDNA3-βgal [27] and LipofectAMINE-2000 (PC-12 and HEK-293 cells) or LipofectAMINE-Plus reagent (MYS cells; both reagents from Invitrogen), following the protocols recommended by the manufacturer. After incubation at 37°C in a 5% CO_2 _atmosphere for 48 h for the PC-12 cells, or 24 h for the HEK-293 and MYS cells, the transfection yield was determined by measuring β-galactosidase activity in cell extracts [28]. The cells were lysed using reporter lysis buffer (Promega). The reaction mixture was made of 50 μl of lysate supernatant, 100 μl of reporter lysis buffer, and 150 μl of 2X-ONPG substrate solution (1.33 mg/ml O-nitro phenyl β-D-Galactopyranoside in 164 mM Na_2_HPO_4_, 36 mM NaH_2_PO_4_, 2 mM MgCl_2 _and 100 mM β-mercaptoethanol). The reaction mixture was incubated at 37°C until development of yellow coloration was apparent (usually within a few hours). Reactions were stopped by the addition of 500 μl of 1 M Na_2_CO_3_. The relative levels of β-galactosidase activity were obtained by determination of the absorbance at 420 nm in a spectrophotometer (Beckman DU500). Parallel assays with cells transfected with pcDNA3 plasmid (Invitrogen) were conducted as negative controls in every experiment, and subtracted from the experimental absorbances to correct for endogenous activities. The experiments were conducted in triplicate. Absorbances were normalized to the values obtained with PEI-pretreated wells.

## Competing interests

The authors declare that they have no competing interests.

## Authors' contributions

A.R.V. performed the experiments comparing the various attachment factors. S.G. and K.L.V.P. performed the PEI dosage, cell number and PEI stability analysis experiments, as well as the NGF differentiation experiments with PC-12 cells. M.J. participated in the experiments with MYS cells. R.P.B. and M.G.G. collaborated as Principal Investigators in this project, participating in the design of the experiments and the writing of the manuscript. R.P.B. performed the experiments with zebrafish retinal explants.
